# Health Resource Utilization Associated with Skeletal-Related Events in Patients with Advanced Prostate Cancer: A European Subgroup Analysis from an Observational, Multinational Study

**DOI:** 10.3390/jcm3030883

**Published:** 2014-07-29

**Authors:** Amit Bahl, Herbert Hoefeler, Ignacio Duran, Guy Hechmati, Cristina Garzon-Rodriguez, John Ashcroft, Vito Lorusso, Prayashi Ghelani, Rachel Wei, Emma Thomas, Diana Lüftner

**Affiliations:** 1 University Hospitals Bristol, Bristol Royal Infirmary, Upper Maudlin St., Bristol, Avon BS2 8HW, UK; 2 Research Centre Ruhr, Pferdebach Street 30, Witten 58455, Germany; E-Mail: hoefeler@forschungszentrum-ruhr.de; 3 Integrated Cancer Centre Clara Campal (CIOCC), Calle Oña 10, Madrid 28050, Spain; E-Mail: ignacioduranmartinez@gmail.com; 4 Health Economics, Amgen (Europe) GmbH, Dammstrasse 23, Zug 6301, Switzerland; E-Mail: hechmati@amgen.com; 5 Institute of Oncology Catalá ICO-IDIBELL, Avenue Gran Via de l’Hospitalet, 199-203, Hospitalet de Llobregat, Barcelona 08908, Spain; E-Mail: cgrodriguez@iconcologia.net; 6 Pinderfields General Hospital, Mid Yorkshire Hospitals NHS Trust, Wakefield WF1 4DG, UK; E-Mail: john.ashcroft@midyorks.nhs.uk; 7 National Cancer Institute IRCCS Giovanni Paolo II, Viale Orazio Flacco 65, 70124, Bari, Italy; E-Mail: vitolorusso@inwind.it; 8 Oncology Institute ASL, Via Antonio Miglietta, Lecce 5 73100, Province of Lecce, Italy; 9 Biostatistics, Ovatech Solutions, 112 Morden Road Abbey, London SW19 2NA, UK; E-Mail: pghelani@amgen.com; 10 Biostatistics, Amgen Inc., 1 Amgen Center Drive, Thousand Oaks, CA 91320, USA; E-Mail: gwei@amgen.com; 11 Scientific Publications, Amgen (Europe) GmbH, Dammstrasse 23, 6301 Zug, Switzerland; E-Mail: embooth@amgen.com; 12 Charité-University Medical Hospital Berlin, Charité Campus Benjamin Franklin, Charitéplatz 1, Berlin D-10117, Germany; E-Mail: diana.lueftner@charite.de

**Keywords:** bone metastases, Europe, health resource utilization, prostate cancer, skeletal-related events

## Abstract

This study aimed to increase the understanding of health resource utilization (HRU) associated with skeletal-related events (SREs) occurring in patients with bone metastases secondary to advanced prostate cancer. A total of 120 patients from Germany, Italy, Spain and the United Kingdom were enrolled in this observational study. They had bone metastases secondary to prostate cancer and had experienced at least one SRE in the 97 days before giving informed consent. HRU data were collected retrospectively for 97 days before enrolment and prospectively for up to 18–21 months. HRU, including the number and duration of inpatient hospitalizations, number of outpatient and emergency department visits and procedures, was independently attributed by investigators to an SRE. Of the 222 SREs included in this analysis, 26% were associated with inpatient stays and the mean duration per SRE was 21.4 days (standard deviation (SD) 17.8 days). Overall, 174 SREs (78%) required an outpatient visit and the mean number of visits per SRE was 4.6 (SD 4.6). All SREs are associated with substantial HRU. Preventing SREs in patients with advanced prostate cancer and bone metastases may help to reduce the burden to both patients and European healthcare systems.

## 1. Introduction

Skeletal-related events (SREs; commonly defined as radiation to bone (RB), pathologic fracture (PF; vertebral (VF) or non-vertebral (NVF)), spinal cord compression (SCC) and surgery to bone (SB)), occur frequently in patients with bone metastases. SREs place a considerable burden on patients, resulting in decreased quality of life and reduced survival [1,2,3]. RB can cause transient increases in pain, and PF can result in persistent pain, reduction in load-bearing capacity and restriction of movement [4,5]. SCC is often a medical emergency and can lead to a temporary or permanent sensory or motor deficit [4], while SB may typically require long inpatient stays that can be traumatic for patients [6,7]. Overall, SREs are associated with reduced patient well-being, both functional and emotional [8], and can result in considerable levels of pain that require strong analgesics [9].

The skeleton is the most common site of metastasis in prostate cancer: 65%–75% of men with advanced disease develop bone metastases [10]. Given that prostate cancer is the second most common form of cancer in Europe and the most common malignancy in men [11], SREs in men with prostate cancer are a significant clinical problem. Furthermore, the health resource utilization (HRU) required to manage these complications places a considerable burden on healthcare systems.

Previous studies have demonstrated the substantial financial burden of SREs in patients with bone metastases from different tumor types, including breast [12], prostate [13,14] and lung [15] cancers in the United States of America (USA). In Europe, Spanish, Portuguese and French studies also found that SREs in patients with breast, prostate or lung cancer were associated with considerable HRU and costs [16,17,18], and demonstrated that the use of hospital resources and the financial burden placed on healthcare systems increases as patients progress to metastatic bone disease and subsequently develop these bone complications [16,18].

Increasing our knowledge of the HRU associated with SREs in patients with prostate cancer through large, prospective studies would assist in improving healthcare planning. In addition, a better understanding of the healthcare resources required for SREs would allow a more accurate assessment of the value of using bone-targeted therapies.

This prospective, observational, multinational study evaluated the HRU associated with SREs in patients with bone metastases/lesions secondary to prostate, breast or lung cancer or multiple myeloma in Canada, Germany, Italy, Spain, the United Kingdom (UK) and the USA. Here we report outcomes for the subset of patients with prostate cancer from the four European countries (Germany, Italy, Spain and the UK).

## 2. Patients and Methods

### 2.1. Patients and Study Design

The methods used in this study have been described previously in detail by Hechmati *et al*. [19]. Investigators described the study to patients when they attended a regularly scheduled clinic visit. Patients who met the eligibility criteria and consented to participate were enrolled. In brief, patients eligible for inclusion were aged 18 years or older with evidence of at least one bone metastasis secondary to prostate cancer, an Eastern Cooperative Oncology Group (ECOG) performance status of 0, 1 or 2, and had experienced at least one SRE in the 97 days before giving informed consent. Patients with a life expectancy of less than 6 months or who were participating in an investigational drug trial for bone metastases were excluded. SREs were classified as RB, PF, SCC or SB.

Investigators collected baseline demographic data and medical history at patient enrolment. HRU data were collected retrospectively for the 97 day period before enrolment and prospectively every 90 days for up to 18–21 months following enrolment. Retrospective and prospective HRU data were obtained from hospital and outpatient records, including clinical and office charts, laboratory and pharmacy records, diaries, microfiches, radiographs, and correspondence. Patients were seen in accordance with their normal medical care; patients were not required to make additional visits to the clinic. If an SRE occurred, HRU data were entered as soon as the investigator was made aware of the SRE. HRU was independently attributed to each SRE by the study investigators. If radiation or surgery to bone was carried out as a result of another SRE (*i.e*., treatment of a primary SRE), the investigator had the option to assign the HRU to the primary SRE. Therefore, SREs determined by investigators to be secondary to a primary SRE were excluded from the HRU analysis.

The primary HRU outcome measures associated with SREs included: (1) inpatient stays: number, duration, reason, type of hospital unit, time spent in each unit; (2) outpatient visits: number, reason; (3) procedures (e.g., imaging, surgery or radiation therapy): number, type; and (4) number of emergency department visits, nursing home/long-term care facility stays and home health visits.

Informed consent was required before collection of patient data. Investigators obtained annual independent ethics committee and institutional review board approval throughout the duration of the study at their individual sites. As such, the ethics committees/institutional review boards of all 70 participating centres approved the study (Amgen study number 20060392) before recruiting patients. The research was carried out in compliance with the Helsinki Declaration.

### 2.2. Statistical Methods

All analyses were descriptive. The proportion of SREs requiring an inpatient stay was calculated as the total number of inpatient-stay days attributed to SREs divided by the total number of SREs of the same type associated with an inpatient stay; if an SRE was associated with multiple inpatient stays, the total duration of all stays was used. All other primary outcome measures were calculated in the same way, as the total number of HRU events attributed to SREs divided by the total number of SREs of the same type.

Outcomes are primarily reported as mean values, rather than medians, because this better describes the total resources used on a population level: information that is required for healthcare policy decisions [20]. Median values are also reported in the figures and where appropriate to illustrate the distribution of outcomes when sample sizes are small or not normally distributed, and to describe the typical HRU for an individual patient.

## 3. Results

### 3.1. Patients

In total, investigators from 45 hospitals enrolled 120 patients with prostate cancer from the four European countries (Germany, *n* = 30; Italy, *n* = 24; Spain, *n* = 21; UK, *n* = 45). The mean age of patients ranged from 69 years to 71 years across the four countries (Table 1). Patients with ECOG status 1 comprised the greatest proportion of patients in each country (46%–60%). Although the patient demographics were generally well balanced across the countries, there was considerable variation in the time since diagnosis of the primary cancer (range of means 41.4–86.3 months); shortest in the UK and longest in Spain (although the mean for Spain was affected by an outlier of 261 months). The mean length of prospective follow-up was in the range of 5.9–8.6 months across the four countries.

### 3.2. SREs

Patients contributed a total of 233 SREs, and SRE rates ranged from 1.6 to 2.4 per patient-year across the four countries (Supplementary Table S1). A total of 11 SREs were excluded from the HRU analysis because they were considered by the investigators to be associated with treatment of another (primary) SRE; the remaining 222 SREs were included in the HRU analysis. RB accounted for 75% of the SREs; 12% of events were SCC, 7% SB and 6% PF.

**Table 1:** Baseline patient demographics and disease history.

Characteristic	Germany (*n* = 30)	Italy (*n* = 24)	Spain (*n* = 21)	UK (*n* = 45)
**Follow-up time, months, mean (SD)**	6.6 (4.9)	8.1 (4.7)	5.9 (4.6)	8.6 (4.7)
**Age, years, mean (SD)**	69.0 (9.5)	70.8 (8.9)	70.0 (9.2)	70.6 (9.1)
**Race, *n* (%)**				
Caucasian	30 (100)	24 (100)	21 (100)	43 (95.6)
Black or African American	0	0	0	2 (4.4)
**ECOG performance status, *n* (%)**				
0	6 (20.0)	9 (37.5)	6 (28.6)	7 (15.6)
1	18 (60.0)	11 (45.8)	10 (47.6)	22 (48.9)
2	6 (20.0)	4 (16.7)	5 (23.8)	16 (35.6)
**Time since primary prostate cancer diagnosis, months**				
Mean (SD)	59.1 (56.9)	47.8 (38.3)	86.3 (70.9)	41.4 (28.6)
Median	41.9	43.8	68.3	36.9
**Time since diagnosis of bone metastasis, months**				
Mean (SD)	16.9 (18.3)	17.8 (16.3)	19.8 (25.1)	19.6 (19.9)^ †^
Median	16.1	15.1	11.1	14.9 ^†^
History of SREs, * *n* (%)	**19 (63.3)**	**12 (50.0)**	**14 (66.7)**	**17 (37.8)**

* Patients who experienced an SRE >90 days before enrolment; † *n* = 44; ECOG—Eastern Cooperative Oncology Group, *n*—Number of patients; SD—Standard deviation, SRE—Skeletal-related event.

### 3.3. Inpatient Stays

**Figure 1:**
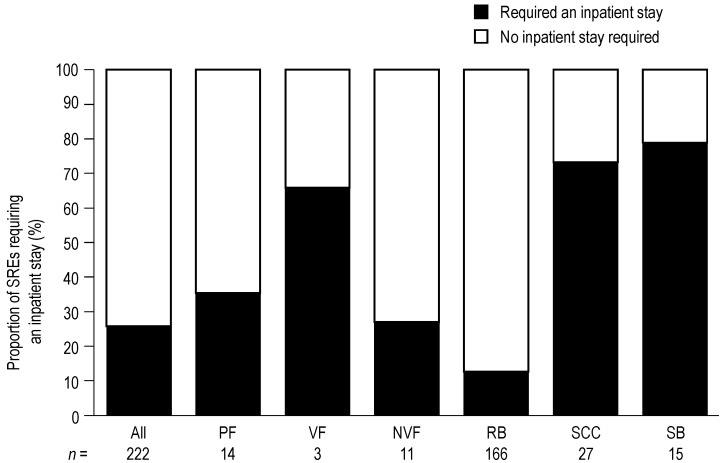
Proportion of skeletal related events (SREs) requiring an inpatient stay. Data show the proportion of SREs requiring an inpatient stay *versus* the proportion of SREs that did not require an inpatient stay. Data for PF are shown first overall, and then by VF and NVF subcategories.

Of the 222 SREs included in the HRU analysis, 58 (26%) required an inpatient stay (Figure 1). RB was the SRE associated with the lowest number of inpatient stays (21 of 166 events; 13%), whereas SB (12 of 15 events; 80%) and SCC (20 of 27 events; 74%) were the SREs most frequently requiring an inpatient stay. Overall, the proportions of SREs requiring an inpatient stay were generally consistent across countries (Supplementary Table S2).

**Figure 2:**
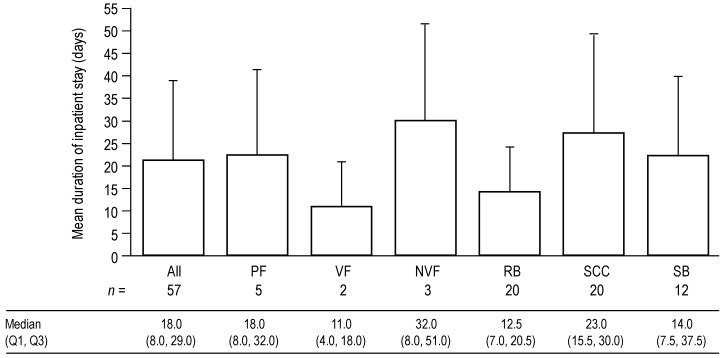
Mean duration of inpatient stays for each type of SRE and for all types of SRE combined. Data shown as mean (+standard deviation) duration of inpatient stays for SREs that required an inpatient stay. Median (Q1, Q3) values are displayed below the graph. If an SRE was associated with multiple inpatient stays, the total duration of all stays was used. Data for PF are shown first overall, and then by VF and NVF subcategories.

For SREs requiring an inpatient stay, the mean duration of inpatient stays was 21.4 days per event (standard deviation (SD) 17.8 days; median (quartile (Q)1, Q3) 18.0 days (8.0, 29.0)) for all types of SRE combined (Figure 2). Inpatient stays were longest for SCC and PF, with mean durations of 27.6 (SD 21.9) and 22.6 (SD 19.2) days, respectively (median 23.0 and 18.0 days, respectively). The mean duration of stay for SB was 22.4 days (SD 17.7); the median duration was 14.0 days. RB was associated with substantial but shorter inpatient stays (mean 14.3 (SD 10.1) days; median 12.5 days).

*n*—Number of SREs in health resource utilization analysis by SRE type; NVF—Non-vertebral fracture; PF—Pathologic fracture; RB—Radiation to bone; SB—Surgery to bone; SCC—Spinal cord compression; SRE—Skeletal-related event; VF—Vertebral fracture.

*n*—Number of SREs associated with inpatient stays by SRE type; NVF—Non-vertebral fracture; PF—Pathologic fracture; Q—Quartile; RB—Radiation to bone; SB—Surgery to bone; SCC—Spinal cord compression; SRE—Skeletal-related event; VF—Vertebral fracture.

The average duration of inpatient stay varied across the countries (range of means 16.3–25.9 days); the mean was lowest in Spain and highest in Italy. In Italy (where seven SREs required inpatient stays), four occurrences of SB with mean stays of 33.0 days contributed to the higher overall mean duration. Similarly, in the UK (where 25 SREs required inpatient stays), 16 occurrences of SCC associated with mean stays of 29.6 days contributed to a larger overall mean for the country (24.0 (SD 22.4) days). The duration of inpatient stay was generally balanced across SRE types in Germany and Spain (Supplementary Table S3).

**Figure 3:**
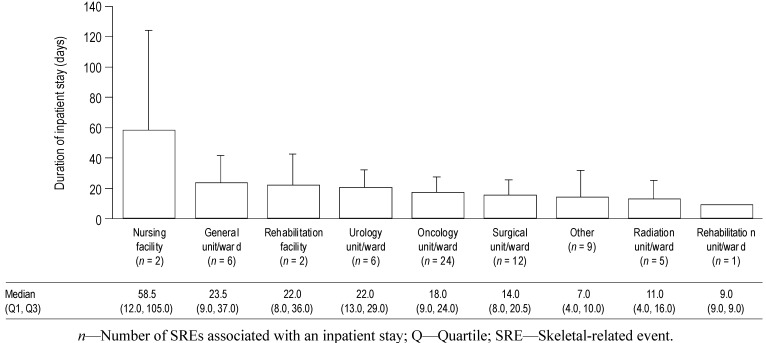
Mean duration of inpatient stays according to healthcare facility type, for all SREs combined. Data shown as mean (+standard deviation) duration of inpatient stays according to healthcare facility type for all SREs combined. Median (Q1, Q3) values are displayed below the graph. If an SRE was associated with multiple inpatient stays, the total duration of all related stays was used.

The duration of inpatient stay according to healthcare facility type for all SREs combined is shown in Figure 3. These data show that the duration of inpatient stay was fairly consistent across most facility types (mean durations approximately 10–20 days), with the exception of nursing facilities. However, there were only two inpatient stays at nursing facilities (both of which were for SCC in the UK), one of 12 days and the other of 105 days, so the high mean duration is the result of a single long stay. Oncology units/wards were the most commonly used facility for inpatient stays (24 SREs required stays) across types of SRE and countries.

*n*—Number of SREs associated with an inpatient stay; Q—Quartile; SRE—Skeletal-related event.

### 3.4. Outpatient Visits

**Figure 4:**
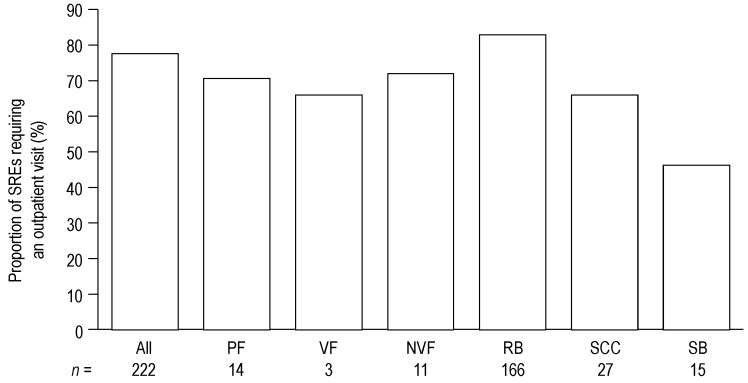
Proportion of SREs requiring an outpatient visit. Data show the proportion of SREs requiring an outpatient visit. Data for PF are shown first overall, and then by VF and NVF subcategories.

Overall, 174 of 222 SREs (78%) required at least one outpatient visit (Figure 4). The proportion of SREs requiring an outpatient visit ranged from 67% to 84% for PF, RB and SCC; in contrast, for SB only 47% of events required an outpatient visit. An outpatient visit was most frequently required for RB (84%) and PF (72%). The proportions of SREs requiring an outpatient visit were similar across the four countries (Supplementary Table S4).

*n*—Number of SREs in the health resource utilization analysis by SRE type; NVF—Non-vertebral fracture; PF—Pathologic fracture; RB—Radiation to bone; SB—Surgery to bone; SCC—Spinal cord compression; SRE—Skeletal-related event; VF—Vertebral fracture.

**Figure 5:**
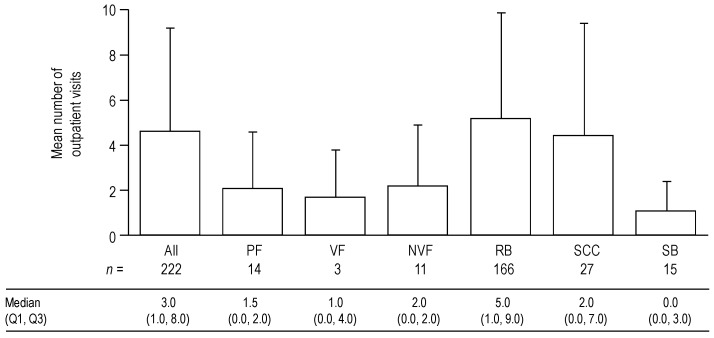
Mean number of outpatient visits for each type of SRE and for all types of SRE combined. Data shown as mean (+standard deviation) number of outpatient visits per SRE. Median (Q1, Q3) values are displayed below the graph. Data for PF are shown first overall, and then by VF and NVF subcategories.

The number of outpatient visits for each type of SRE is shown in Figure 5. Across all countries and SRE types, the mean number of outpatient visits per SRE was 4.6 (SD 4.6) (median (Q1, Q3) 3.0 (1.0, 8.0)). RB was associated with the highest number of outpatient visits (mean 5.2 (SD 4.7)) followed by SCC (mean 4.4 (SD 5.0)); SB was associated with the lowest number (mean 1.1 (SD 1.3)). The number of outpatient visits per SRE was higher in Germany and Spain (means 6.3 (SD 5.6) and 6.6 (SD 4.7), respectively) than in Italy and the UK (means 3.9 (SD 3.5) and 3.2 (SD 3.7), respectively). A lower number of outpatient visits for RB in Italy and the UK was the main contributor to these differences (Supplementary Table S5).

*n*—Number of SREs included in the health resource utilization analysis by SRE type; NVF—Non-vertebral fracture; PF—Pathologic fracture; Q—Quartile; RB—Radiation to bone; SB—Surgery to bone; SCC—Spinal cord compression; SRE—Skeletal-related event; VF—Vertebral fracture.

### 3.5. Procedures

**Figure 6:**
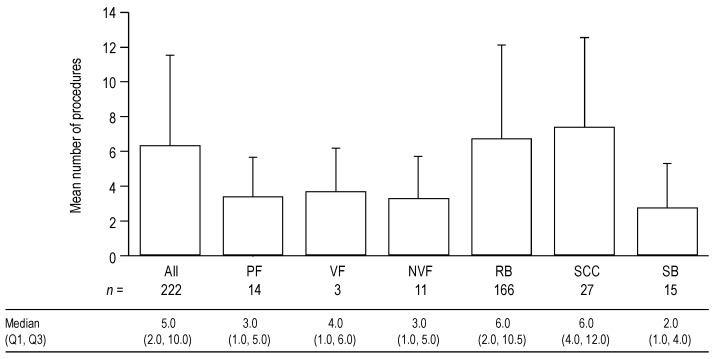
Mean number of procedures for each type of SRE and for all types of SRE combined. Data shown as mean (+standard deviation) number of procedures per SRE. Median (Q1, Q3) data are displayed below the graph. Data include outpatient visits, overnight stays and emergency department visits. Data for PF are shown first overall, and then by VF and NVF subcategories.

All of the SREs recorded required a procedure, with the exception of RB events, for which additional procedures were reported in 95% of cases. Owing to the observational nature of the study, it is probable that in the 5% of cases in which a procedure was not reported, a procedure was performed but was not recorded correctly. The number of procedures for each type of SRE is shown in Figure 6. For all SRE types combined, the mean number of procedures per SRE was 6.3 (SD 5.2) (median (Q1, Q3) 5.0 (2.0, 10.0)). SCC was the SRE type associated with the highest number of procedures (mean 7.4 (SD 5.2)), followed by RB (mean 6.7 (SD 5.4)).

*n*—Number of SREs included in the health resource utilization analysis by SRE type; NVF—Non-vertebral fracture; PF—Pathologic fracture; Q—Quartile; RB—Radiation to bone; SB—Surgery to bone; SCC—Spinal cord compression; SRE—Skeletal-related event; VF—Vertebral fracture.

The number of procedures for the other SRE types was similar (means 2.7–3.7 per SRE). The overall number of procedures per SRE was higher in Germany and Spain (means 9.6 (SD 6.5) and 8.7 (SD 3.2), respectively) than in Italy and the UK (means 5.0 (SD 5.0) and 4.1 (SD 4.0), respectively). This pattern was also seen for the number of procedures per RB event (means 11.2 (SD 6.0) and 8.9 (SD 3.0) for Germany and Spain, respectively, compared with 5.8 (SD 4.2) and 3.2 (SD 3.2) for Italy and the UK, respectively).

For all 222 SREs included in the HRU analysis, the main procedures carried out were: external beam radiation (mean 3.0 (SD 3.9) occurrences per SRE); intensity modulation radiotherapy (2.0 (SD 4.8) per SRE); X-ray (0.3 (SD 0.7) per SRE); computed tomography (0.2 (SD 0.5) per SRE); magnetic resonance imaging (0.2 (SD 0.4) per SRE); physical examination (0.2 (SD 0.5) per SRE); and laboratory assessment (0.1 (SD 0.6) per SRE). Other types of procedure that were required less frequently (<0.1 occurrences per SRE) included surgery to bone in the extremities, surgery to the spine, use of radionucleotides, radionuclide scanning, transfusion, ultrasound and wound care.

### 3.6. Emergency Department and Home Health Visits, and Nursing Home/Long-term Care Facility Stays

Of the 222 SREs included in the analysis, three (1.4%) required a home health visit and eight (3.6%) needed an emergency department visit, of which seven were associated with a single visit, and one (a SCC event) was associated with three or more visits. SB was the only SRE that did not require an emergency department visit. No SREs required a nursing home/long-term care facility stay.

## 4. Discussion

This is the first study to prospectively analyze HRU associated with SREs in patients with advanced prostate cancer. The results show that, overall, more than one-quarter of SREs required inpatient stays, and that these were generally lengthy, with a mean duration of approximately 21 days across all SREs and countries. This study also shows that SREs were associated with a large number of outpatient visits and procedures in patients with prostate cancer. These prospective and retrospective data confirm that SREs in patients with prostate cancer require considerable HRU, as reported in two previous retrospective European studies [17,21].

RB is the most commonly occurring SRE in prostate cancer [22,23], despite being the SRE least frequently associated with inpatient stays (13% of SREs), and this event is still likely to contribute significantly to SRE-related hospitalizations. Furthermore, when RB was associated with inpatient stays, the duration of these stays was substantial (mean 14.3 days). The majority of RB events required an outpatient visit (83.7%), and this SRE was also associated with the highest number of outpatient visits per SRE. In addition, RB was associated with one of the highest mean number of procedures per SRE. The high frequency of these events combined with their requirement for HRU suggests that they contribute significantly to the costs of patient care. Two retrospective studies of patients with prostate cancer in the USA found RB to be the greatest contributor to the medical care costs associated with SREs [13,14].

Although they occur less frequently [22,23], SB and SCC were the SREs most likely to require inpatient stays (80% and 74% of events required hospitalization, respectively). These stays probably led to substantial costs, as demonstrated by another study which found that hospitalization accounted for the largest proportion of SRE-related expenditure [17]. The high proportion of inpatient stays seen for SB explains why these events were associated with the fewest outpatient visits per SRE. Surgery stabilizes the fractures; therefore subsequent outpatient support is often not required. In contrast, SCC was associated with a high number of outpatient visits per SRE, reflecting the need for continued care following the initial acute event.

Overall, patterns of HRU were consistent across the countries. However, some variation in the numbers of outpatient visits and procedures was seen across the four countries, with higher numbers in Germany and Spain, and lower numbers in Italy and the UK. These differences can probably be attributed to a higher number of RB procedures in Germany and Spain compared with Italy and the UK. Differences in clinical practice among the countries may help to explain this variation; specifically, multiple-fraction radiation sessions are more commonplace in mainland Europe [17,24], while single-fraction radiation is used more often in the UK [25,26].

One limitation of the study is that the follow-up times were shorter than planned and the numbers of patients enrolled were lower than anticipated. Slow recruitment into the study and early withdrawal due to patient death were the main contributors to this shorter-than-planned median follow-up period; patient withdrawal for other reasons may also have been a factor. The length of follow-up varied across the countries. The study period was defined as 30 months following enrolment of the first patient; therefore the potential length of follow-up varied according to when the centers enrolled their patients. Variations in clinical practice and the availability of alternative trials offering active therapies may also have affected the length of follow-up. The sample sizes for SB and SCC were limited and may not have been sufficient to provide a generalizable HRU estimation. Similarly, the applicability of estimations of the durations of inpatient stays may have been limited by the small sample sizes.

The study may have underestimated the overall HRU associated with bone metastases and SREs in clinical practice because of the exclusion of patients with a short life expectancy (less than 6 months) and poor ECOG performance status (3 or 4). Furthermore, pain was not defined as an SRE, although this frequent complication of inadequately treated bone metastases could have led to additional HRU, and requirement for analgesics. Accurate assessment of resource use associated with home health visits/nursing home stays was difficult because this information is often not relayed back to the main hospital and is driven more from a primary care setting. Therefore, some data were not available to investigators at all study sites. It should also be noted that the incidence of SREs reported in this study is not representative of the real-world distribution of SREs because enrolment was driven by the index SRE recruitment cells. Furthermore, we did not enrol an equal number of patients with ECOG performance status 0, 1 and 2, and as such, we cannot conclude whether patients with a worse performance status would have required greater HRU. However, HRU was directly attributed to SREs by investigators, and was therefore only assigned when it was considered to be directly related to the SRE, rather than the underlying disease.

Despite these limitations, it can be concluded that all SRE types were associated with substantial HRU and that the patterns of HRU were generally consistent across all countries studied. The level of HRU associated with SREs identified in this study is likely to be associated with a considerable economic impact. Optimal therapies that help to reduce the incidence of SREs may have an important role to play in reducing HRU and the associated economic burden in patients with advanced prostate cancer. The intravenous bisphosphonate, zoledronic acid, has been the mainstay of treatment for the prevention of SREs in patients with bone metastases secondary to advanced prostate cancer [27]. Recently, denosumab, the monoclonal antibody against RANK ligand, has been approved for the reduction of SREs in patients with bone metastases arising from solid tumors [28], and has demonstrated superiority over zoledronic acid in the prevention of first and subsequent SREs in patients with prostate cancer [22].

## 5. Conclusions

This is the largest European study including prospectively collected data on the HRU associated with SREs in prostate cancer; therefore, these data give a valuable indication of the probable impact of SREs. In summary, the results show that all SREs are associated with substantial HRU in patients with prostate cancer in Europe. Preventing SREs in patients with advanced prostate cancer and bone metastases via effective bone-targeted agents may help to reduce the HRU burden and associated costs in the European healthcare system.

## Supplementary Files

Supplementary File 1
