# Supplementary material for: Health Resource Utilization Associated with Skeletal-Related Events in Patients with Advanced Prostate Cancer: A European Subgroup Analysis from an Observational, Multinational Study
Source: J Clin Med. 2014 Jul 29;3(3):883–96. doi: 10.3390/jcm3030883 (PMC4449648; doi:10.3390/jcm3030883)
Supplement: Supplementary File 1 [file jcm-03-00883-s001.pdf]

Supplementary Information

Table S1. SRE rates per patient-year according to country and SRE type.

SRE type	Germany (n = 30)	Italy (n = 24)	Spain (n = 21)	UK (n = 45)
All SREs	2.4	1.6	2.4	2.4
PF	0.2	0.1	0.1	0.1
RB	1.8	1.2	2.1	1.7
SCC	0.1	0.1	0.2	0.4
SB	0.2	0.2	0.0	0.2

n—Number of patients; PF—pathologic fracture; RB—radiation to bone; SB—surgery to bone; SCC—spinal cord compression; SRE—skeletal-related event.

Table S2. Proportions of SREs requiring an inpatient stay by SRE and by country.

SRE type, *n* (%)	Germany	Italy	Spain	UK
All SREs	16 (29)	7 (21)	9 (25)	26 (27)
PF	2 (40)	0 (0)	1 (50)	2 (50)
RB	10 (23)	2 (8.0)	6 (19)	3 (4.5)
SCC	1 (33.3)	1 (50)	2 (67)	16 (84)
SB	3 (75)	4 (100)	-	5 (71)

n—Number of SREs requiring an inpatient stay; PF—pathologic fracture; RB—radiation to bone; SB—surgery to bone; SCC—spinal cord compression; SRE—skeletal-related event.

Table S3. Duration of inpatient stays (in days) by SRE and by country.

SRE type	Germany (n = 16)		Italy (n = 7)		Spain (n = 9)		UK (n = 25)	
	Mean (SD)	Median (Q1, Q3)	Mean (SD)	Median (Q1, Q3)	Mean (SD)	Median (Q1, Q3)	Mean (SD)	Median (Q1, Q3)
All SREs	18.3 (13.6)	15.5 (8.5, 26.0)	25.9 (14.3)	21.0 (13.0, 43.0)	16.3 (11.2)	15.0 (8.0, 19.0)	24.0 (22.4)	18.0 (8.0, 29.0)
PF	18.0 (19.8)	18.0 (4.0, 32.0)	0.0 (0.0)	0.0 (0.0, 0.0)	18.0 (0.0)	18.0 (18.0, 18.0)	29.5 (30.4)	29.5 (8.0, 51.0)
RB	16.7 (9.0)	17.0 (10.0, 23.0)	14.0 (9.9)	14.0 (7.0, 21.0)	14.5 (12.1)	11.5 (7.0, 19.0)	2.0 (0.0)	2.0 (2.0, 2.0)
SCC	15.0 (0.0)	15.0 (15.0, 15.0)	21.0 (0.0)	21.0 (21.0, 21.0)	21.0 (14.1)	21.0 (11.0, 31.0)	29.6 (23.9)	26.0 (16.0, 31.0)
SB	24.7 (27.2)	11.0 (7.0, 56.0)	33.0 (14.4)	37.5 (22.5, 43.5)	-	-	12.6 (10.0)	8.0 (7.0, 15.0)

n—Number of SREs associated with inpatient stays by SRE type; PF—pathologic fracture; RB—radiation to bone; SB—surgery to bone; SCC—spinal cord compression; SRE—skeletal-related event.

Table S4. Proportions of SREs requiring an outpatient stay by SRE and by country.

SRE type, *n* (%)	Germany	Italy	Spain	UK
All SREs	41 (75)	28 (82)	28 (78)	77 (79)
PF	4 (80)	3 (100)	1 (50)	2 (50)
RB	35 (81)	23 (92)	26 (84)	55 (82)
SCC	2 (67)	1 (50)	1 (33)	14 (74)
SB	0 (0.0)	1 (25)	-	6 (86)

n—Number of SREs requiring an outpatient stay; PF—pathologic fracture; RB—radiation to bone; SB—surgery to bone; SCC—spinal cord compression; SRE—skeletal-related event.

Table S5. Number of outpatient visits per SRE, by SRE and by country.

SRE type	Germany (*n* = 55)		Italy (*n* = 34)		Spain (*n* = 36)		UK (*n* = 97)	
	**Mean (SD)**	**Median (Q1, Q3)**	**Mean (SD)**	**Median (Q1, Q3)**	**Mean (SD)**	**Median (Q1, Q3)**	**Mean (SD)**	**Median (Q1, Q3)**
All SREs	6.3 (5.6)	7.0 (0.0, 11.0)	3.9 (3.5)	4.5 (1.0, 5.0)	6.6 (4.7)	8.0 (1.0, 11.0)	3.2 (3.7)	2.0 (1.0, 5.0)
PF	1.2 (0.8)	1.0 (1.0, 2.0)	4.0 (4.4)	2.0 (1.0, 9.0)	2.0 (2.8)	2.0 (0.0, 4.0)	1.8 (2.4)	1.0 (0.0, 3.5)
RB	7.3 (5.4)	8.5 (2.5, 11.0)	4.6 (3.4)	5.0 (1.0, 5.0)	7.2 (4.5)	8.0 (2.0, 11.0)	3.2 (3.6)	1.5 (1.0, 5.0)
SCC	8.7 (7.6)	12.0 (0.0, 14.0)	2.5 (3.5)	2.5 (0.0, 5.0)	3.7 (6.4)	0.0 (0.0, 11.0)	4.1 (4.5)	2.0 (0.0, 6.0)
SB	0.0 (0.0)	0.0 (0.0, 0.0)	0.3 (0.5)	0.0 (0.0, 0.5)	-	-	2.1 (1.2)	3.0 (1.0, 3.0)

n—Number of SREs included in the health resource utilisation analysis; PF—pathologic fracture; RB—radiation to bone; SB—surgery to bone; SCC—spinal cord compression; SRE—skeletal-related event.

© 2014 by the authors; licensee MDPI, Basel, Switzerland. This article is an open access article distributed under the terms and conditions of the Creative Commons Attribution license (http://creativecommons.org/licenses/by/3.0/).
